# In Vivo Imaging of Biodegradable Implants and Related Tissue Biomarkers

**DOI:** 10.3390/polym13142348

**Published:** 2021-07-17

**Authors:** Leon Riehakainen, Chiara Cavallini, Paolo Armanetti, Daniele Panetta, Davide Caramella, Luca Menichetti

**Affiliations:** 1 Institute of Clinical Physiology, National Research Council (IFC-CNR), 56124 Pisa, Italy; chiara.cavallini@ifc.cnr.it (C.C.); paolo.armanetti@ifc.cnr.it (P.A.); daniele.panetta@ifc.cnr.it (D.P.); luca.menichetti@ifc.cnr.it (L.M.); 2 College of Medicine, Sant’Anna School of Advanced Studies, 56127 Pisa, Italy; 3 Specialization School in Radiodiagnostics, University of Pisa, 56100 Pisa, Italy; davide.caramella@unipi.it

**Keywords:** biodegradable implants, implant imaging, multimodal imaging, biomarkers, computed tomography (CT), positron emission tomography (PET), ultrasound (US), photoacoustic imaging (PAI), magnetic resonance imaging (MRI)

## Abstract

Non-invasive longitudinal imaging of osseointegration of bone implants is essential to ensure a comprehensive, physical and biochemical understanding of the processes related to a successful implant integration and its long-term clinical outcome. This study critically reviews the present imaging techniques that may play a role to assess the initial stability, bone quality and quantity, associated tissue remodelling dependent on implanted material, implantation site (surrounding tissues and placement depth), and biomarkers that may be targeted. An updated list of biodegradable implant materials that have been reported in the literature, from metal, polymer and ceramic categories, is provided with reference to the use of specific imaging modalities (computed tomography, positron emission tomography, ultrasound, photoacoustic and magnetic resonance imaging) suitable for longitudinal and non-invasive imaging in humans. The advantages and disadvantages of the single imaging modality are discussed with a special focus on preclinical imaging for biodegradable implant research. Indeed, the investigation of a new implant commonly requires histological examination, which is invasive and does not allow longitudinal studies, thus requiring a large number of animals for preclinical testing. For this reason, an update of the multimodal and multi-parametric imaging capabilities will be here presented with a specific focus on modern biomaterial research.

## 1. Introduction

Because of the invasive nature of implantation practice and associated organism reactions to the presence of foreign materials, imaging has been used as a tool to monitor the patient’s condition ever since the discovery of X-rays in 1895. With the development of technologies and the increase in variety, the possible aspects and options for imaging have increased, allowing the visualization not only of structural condition, but also biological reactions and interactions. 

Currently, implants used in orthopaedics, dentistry, reconstructive and cosmetic surgery use a large variety of materials, including permanent implants made of polymers (polyurethane, polyethylene, polypropylene, polymethylmethacrylate etc.) [1], ceramics (aluminium oxide, zirconium oxide, carbon-silicon etc.) and metals (titanium, stainless steel, gold, cobalt-chromium etc.) [2] However, it is unavoidable that most, if not all of the used materials have side effects, requiring additional treatment or even removal surgeries [3]. In some cases, complications such as patient discomfort, osteopenia due to stress shielding and chronic inflammatory reaction could be reduced or avoided if the implants would disappear after their effect is no longer required. For this reason, the development and application of biodegradable implants has become an attractive topic in implantology. This requires high quality testing and trials to confirm the safety and quality of these implants. One of these requirements is to have adequate imaging capabilities to follow up tissue healing and implant degradation in vivo.

Because of the ongoing diversification and narrow specialization in the field of science, the available information has become increasingly diverse and complicatedly interlinked. Concerns have been raised about the insufficient mutual understanding of the needs and capabilities among the specialists in the related fields such as medicine, biotechnology and imaging. As a result, the inadequate combinations of imaging modalities and targets have resulted in studies not being performed to their full potential. This review provides a detailed overview of the non-invasive imaging techniques commonly used in preclinical studies: computed tomography (CT), positron emission tomography (PET), ultrasound (US), PAI (photoacoustic imaging) and MRI (magnetic resonance imaging). The advantages and limitations of these modalities are evaluated for imaging the available biodegradable metallic, ceramic and polymer implants and the related tissue healing processes through targeting biomarkers of bone regeneration, angiogenesis and inflammation.

### 1.1. Background

With the scientific progress and development of new materials, by the 20th century, a variety of new substances and multi-phase materials were found and developed, such as alloys based on titanium, zirconium oxide, cobalt-chromium, nickel-chromium, stainless steels, polymers etc. [2] This had allowed the dental and bone implants to become a standard approach in defect repair and replacement. 

With the improvement of the average quality of life and subsequent increase in requirements, attention was turned towards specially designed biodegradable implants that would allow faster healing, fewer side effects and better comfort. Despite the first biodegradable materials such as proteins (silk and collagen used in sutures) and metals (magnesium wires and plates) knowingly used for over a century, only recently has the scientific methodology been developed enough to allow a sufficiently reliable and comprehensive analysis of their effects on the living body.

**Figure 1:**
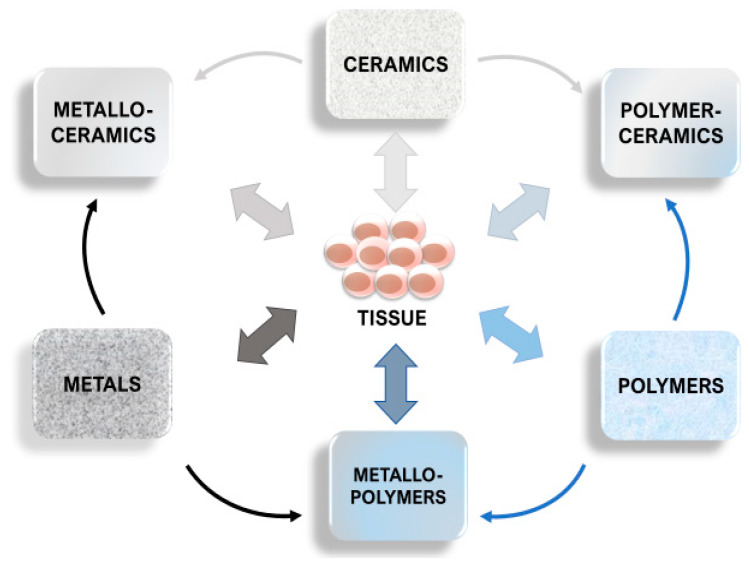
Implant types by materials. While most research focuses on tissue interaction with a single type of material, currently, the variety of available compound materials allows us to modify the material properties and their behaviour according to requirements.

**Figure 2:**
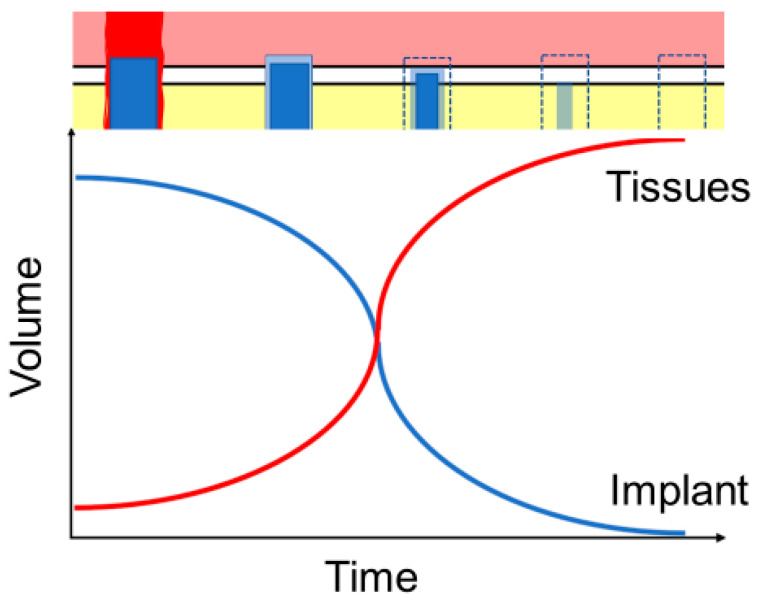
Ideal implant degradation and tissue regrowth dynamics against time. The rate of implant degradation has to be balanced to correspond to tissue regeneration, so the lost implant volume and mechanical strength are compensated by new tissue until the implant is completely degraded and absorbed.

Natural and synthetic polymers (proteins and poly[hydroxyl acids]), ceramics (bioactive glass, tricalcium phosphate, hydroxyapatite), metals (Mg, Fe, Zn) and their composites are known to be biodegradable [4] and already see a limited use in implantology (Figure 1). Their ability to perform their desired function without causing any local or systemic adverse response in the recipient is known as biocompatibility [5]. For an implant to perform its desired function, successful integration with the surrounding tissue is required. That, in turn, is a complex process involving aspects of wound healing, cell signalling, proliferation, adhesion, and growth, which need to be coordinated between multiple tissue types. In biodegradable implants, the process of corrosion is one of the main measures of biocompatibility, different from the traditional implants, where the corrosion is seen as a flaw. With these implants, in addition to the by-products of the degradation being produced, the surrounding tissues are continuously growing while the volume of the implants is correspondingly reduced. The balanced rate of degradation and growth are essential to ensure the correct maintenance of the mechanical integrity, especially in load-bearing structures such as bones [4] (Figure 2).

Based on the above, the potential targets for clinical and research imaging of biodegradable implants are: (i) immediate and long-term tissue and cell responses; (ii) tissue regeneration; (iii) implant integration (implant–tissue interface); and (iv) changes in implant structure. The biological processes have specific characteristics that are known as biomarkers [6] which can be used to evaluate the changes and to serve as targets for biomedical imaging. It is necessary to follow the degradation of the implants in vivo from mechanical, chemical and biological standpoints to ensure that they remain safe and functional through the whole duration of the treatment.

### 1.2. Tissue Response 

The damage to the living tissue and subsequent healing are closely associated with the inflammation process that is controlled by the immune system. While the actual details of cellular and biochemical reactions can differ depending on the severity and the site of the trauma, in general the inflammatory response can be separated into: (1) recognition of the harm by cell receptors (danger-associated molecular patterns of pattern recognition receptors); (2) activation of inflammatory pathways (such as intracellular signalling pathways that involve messenger molecules such as cytokines and receptors); (3) release of inflammation markers (mostly cytokines, proteins and enzymes involved in inflammatory cell activity); and (4) recruitment of inflammatory cells (macrophages, monocytes, neutrophils, lymphocytes, mast cells and platelets) [7]. In the presence of biodegradable implants, these responses are affected by the chemical and mechanical interaction between the tissues and the implants and released degradation products.

Blood vessel formation, or angiogenesis, is a crucial part of trauma healing, ensuring the necessary transport of the metabolic molecules and the formation of the regenerated tissues. This directly influences the biochemical processes associated with damage and subsequent healing. When dealing with the biodegradable implants, blood vessels are also involved in the disposal of the degradation products, such as in the case of metal ions [8]. 

**Figure 3:**
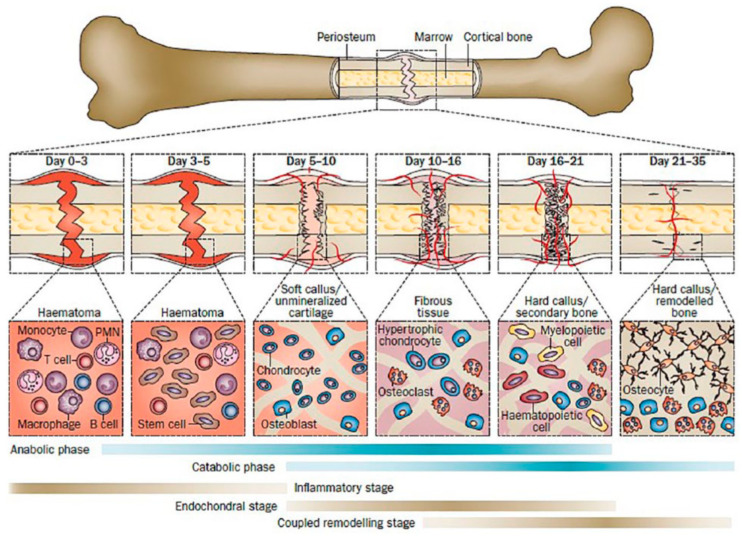
Healing stages of mouse closed femur fracture fixed with an intramedullary rod. Adapted with permission from Einhorn et al. (2015) [10] Copyright Springer Nature, 2014.

Bone regeneration in the context of implants refers to the absorption of damaged tissue and the growth of new tissue around the bone implant. The involved pathways of chondrocyte, osteoclast and osteoblast activity regulation are highly complex, involving specific and non-specific tissue healing cell signals which are directly linked to hematoma from damaged blood vessels and inflammatory reactions [9]. Physically, the bone regeneration can be regarded as the growth of the bone volume to compensate for the loss caused by the damage and the gradual substitution of the degrading implant. Figure 3 shows the sequence of the bone healing stages, adapted from Einhorn et al. (2015) The efficiency of bone healing, especially at the remodelling phase, affects the changes in bone morphology and the osseointegration quality of the implant. That is especially important for the degrading implants, whose volume changes over time, because it directly affects the long-term implant stability and the quality of life of the patients.

### 1.3. Effects of Implant Structure

Because they are essentially designed to become a part of the human body, both chemical and mechanical properties of the biodegradable implants have to fulfil strict requirements. They need to be biochemically neutral, non-toxic and must not trigger adverse reactions. At the same time, they must have a controlled, gradual rate of degradation while maintaining structural integrity, which is needed to allow the tissues to regenerate and compensate for the lost implant volume without the danger of damage from fragmentation. 

**Figure 4:**
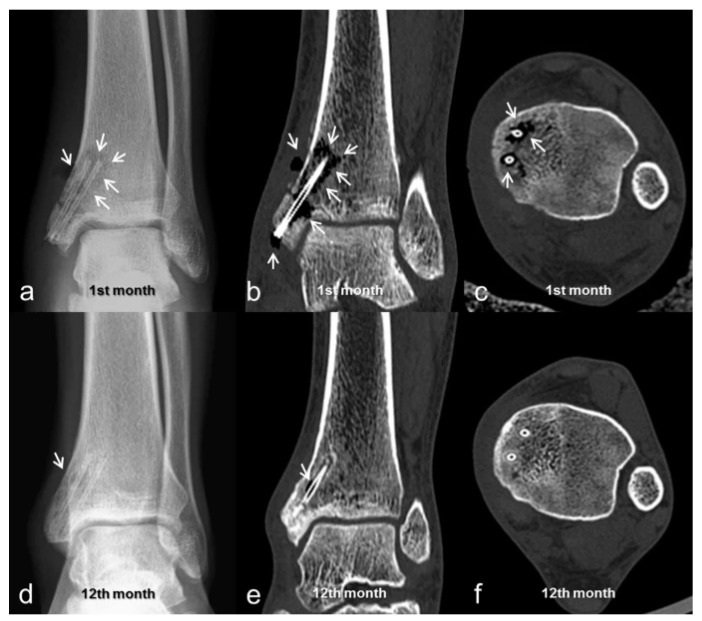
Isolated medial malleolar fracture treated with biodegradable magnesium screws (MAGNEZIX^®^ CS; Syntellix AG, Hanover, Germany). (**a**–**c**) taken 1 month after implantation; (**d**–**f**) on 12th month after implantation. (**a**,**d**) are radiographs; (**b**,**c**,**e**,**f**) are taken using CT. White arrows point at gas formation around implant. Reproduced from May et al. (2020) [15]. Copyright Springer Nature, 2020.

Chemical properties of implants are mostly influenced by the material they are made of. A major factor is the chemical reaction of the implanted material with the surrounding tissues, and corresponding changes in local biochemistry. The resulting changes in cell activity that affect the whole process of wound healing and tissue remodelling can be material-specific. By making use of alloys or otherwise mixed materials, the possible variables affecting degradation and interaction with the tissues can be manipulated with high flexibility and precision. Notable examples for this approach is the use of calcium, zinc, aluminium and various rare metals to adjust the degradation properties of magnesium implants [4,11] and hydroxyapatite composites that include collagen, poly(l-lactide) (PLLA), polydiolcitrates, polyvinyl alcohol or other polymers [12,13,14]. Figure 4 presents an example of clinical application of a specially designed compression screw from MgYREZr alloy (magnesium with yttrium, rare earth metal, and zirconium) and surface treatment to possess an oxide film and to combine both material and production methods to optimize their performance [15]. 

Mechanically, implants differ according to production methods, geometry and surface treatment. Production methods decide the microstructure—arrangements of the atoms, density, purity, distribution of additives etc. Depending on the material, these variables can have significant effects on the physical properties of the final product, such as absorption, scattering and attenuation of photons. Geometry, or the shape, of the implants is diverse—both due to specialized uses and also patent analogues from competing manufacturers. In the case of biodegradable implants, geometry also changes during the degradation process. 

For imaging applications, it is necessary to consider the possible effects of the presence of irregular and thin shapes that can be difficult to recognize. Surface treatment, as the border between the bulk of the implant and the body, has the principal role of interaction with the tissues. Since this decides the success of implant integration, it is the primary focus of imaging, be it in the clinical or research approach. The use of coatings to promote the integration, reduce the adverse reactions and regulate the degradation is increasing, with notable examples being magnesium implants coated with hydroxyapatite, polymers, oxide layers etc [16,17]. A variety of biodegradable materials from all three types (ceramics, polymers, metals) that have been reported in the literature are listed in Table 1.

## 2. Features of the Imaging Techniques 

### 2.1. Computed Tomography

Computed tomography, being the three-dimensional version of the conventional planar X-ray scan, relies on the same principles of using electromagnetic radiation (photons) and the difference in its attenuation rate by different matter. As photons interact with the surrounding matter, they can be absorbed by a photoelectric effect, or undergo scattering through incoherent (Compton) or coherent (Rayleigh) scattering.

In the photoelectric effect, the incident photon is absorbed by the atom while displacing the electron from its shell, which creates the contrast in the rays absorbed by the matter, creating the image. This effect is dependent on electron binding energies, as the lower binding energy allows lower energy photons to interact, and the probability is generally proportional according to formula Z^3^/E^3^ (Z = atomic number; E = photon energy). Except for the absorption edge points in the absorption spectrum of the substance, where there is a sharp rise in absorption coefficient as the energy increases, and the energy of the photon becomes equal to the energy of the electron shell. The combination of the scattering and absorption coefficients is represented as mass absorption coefficient (in cm^2^/g). In radiology, the mass absorption coefficient has little practical use, instead these values are multiplied by density to obtain linear absorption coefficient (in cm^−1^). Table 2 provides selected values of linear absorption coefficients based on densities from Table 1 for biodegradable implant bulk materials and references (titanium, skeletal muscle and cortical bone) across the range of clinical CT photon energies. Depending on microstructure and alloy composition, the density and therefore the resulting linear absorption coefficient values can significantly differ. For this, Table A1 in Appendix A provides raw calculated total attenuation coefficients without coherent scattering for most materials and the energy range. Beam hardening is the most common implant-related artefact, created when lower energies of the polychromatic X-ray beam are absorbed while letting the high energy photons to pass through, thus distorting the calculation of thickness-based attenuation data [33]. That can be reduced by achieving a monochromatic beam (usually simulated by dual energy CT) or by increasing photon energies (increased radiation dose). While the clinical CT photon energies are between 5 (mammography) and 150 (abdominal) keV [34], the actual usable energies are further limited by the volume (and density) of the imaging target and technical limitations of the available equipment.

Therefore, to achieve a clear image of an implant, there needs to be sufficient contrast between it and the surrounding tissues while staying within the acceptable photon energy range and ensuring that the attenuated photons do not go below the detector minimum detection range. The polymers, bioengineered implants and grafts are often indistinguishable from the surrounding tissues because of the similar absorption properties. To image these, using CT requires special approach to enhance the contrast, such as alloying the implant material to create composites with desirable properties or using surface coating. The widely spread titanium, stainless steel and zirconium oxide implants have absorbance coefficients significantly higher than those of the body tissues, so they are easily distinguishable. However, the presence of metals produces significant artefacts that reduces the quality of the obtained images [35,36,37]. At the same time, the developing magnesium-based biodegradable implants are less affected by this effect [38]. 

**Figure 5:**
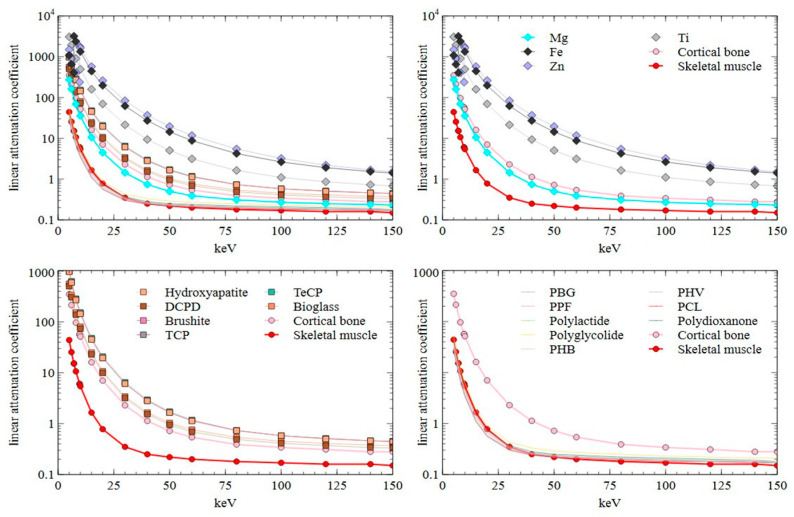
Plotted linear attenuation coefficient data of biodegradable implant materials and cortical bone and skeletal muscle. PBG = poly(γ-benzyl-L-glutamate); PPF = polypropylene fumarate; PHB = polyhydroxybutyrate; PHV = polyhydroxyvalerate; PCL = polycaprolactone.

Despite the limitations, CT is a reliable and trusted method for imaging bone pathologies, structural changes and with the use of contrast agents, vascularisation. According to data in Table 2 (graphically represented in Figure 5), only a few implants have a linear attenuation similar to cortical bone (such as magnesium and ceramics), and even then, most materials can provide sufficient border contrast. That, and artefact reduction, is achieved by using dual energy protocols [39,40]. At the same time, CT is often necessary for surgery planning and is helpful in detecting implant-related complications including inflammations [41] and osteolysis [42]. 

### 2.2. Positron Emission Tomography

Positron emission tomography uses positrons that are emitted during beta plus (β^+^) decay of radionuclides that are the signalling components of the radiotracers. The emitted positron interacts with a surrounding electron and the mutual annihilation reaction produces two photons of gamma energy spectrum (511 keV) that move in opposite directions at a 180° angle from each other. The photons are detected, and the annihilation event location is reconstructed using corresponding algorithms. 

**Figure 6:**
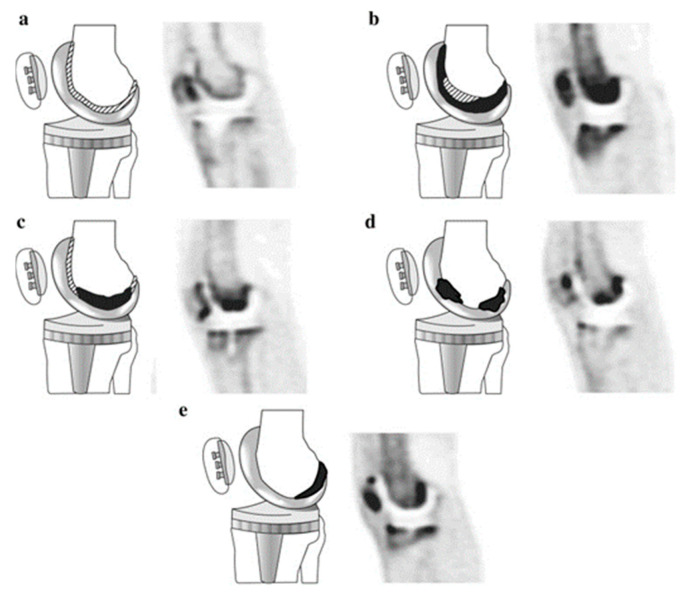
Diagrams and representative PET images showing the five types of ^18^F-NaF uptake pattern in the femoral component of knee prostheses. Black areas represent areas of severely increased uptake and shaded areas represent slightly increased uptake of radiotracer. Adapted with permission from Son et al. (2016) [50] Copyright Springer Nature, 2016.

While a metabolically inactive implant would have little to no interaction with the radiotracer, PET allows us to specifically image the metabolic activity of chosen pathways, which is indispensable for the evaluation of the tissues affected by the implantation. For this reason, the targets of the imaging are metabolites and pathways that are relevant for the study focus. In the case of biodegradable implants, this involves their interaction with the surrounding tissues during the inflammatory stage and the following processes of tissue regeneration, implant integration, degradation and replacement. Osseointegration of bone implants and the substitution of degraded material with new bone can be monitored through the increase of calcium, for example through the use of ^18^F that binds to hydroxyapatite, thus creating fluorapatite [43]. Inflammation associated with trauma healing and the presence of foreign material in the body is routinely imaged by quantifying the increase in glucose metabolism by using fluorodeoxyglucose (FDG) [44]. Wound healing and regeneration of the surrounding tissues can be associated with the growth of blood vessels and the activity of associated integrins such as α_V_β_3_ [45,46] and α_V_β_5_ [47], which are targeted by tracers based on RGD-peptides (e.g., ^68^Ga-NODAGA-RGD [47]). There are also groups of biomarkers, such as matrix metalloproteinases (MMPs, a family of endopeptidases), that have a varied role in biochemical activity, for example MMP-9 has been shown to be associated with early extracellular matrix (ECM) re-organization [48], and MMP-12 is linked to macrophage activity and vascular pathologies [49]. In Figure 6, from a study of asymptomatic patients with hip and knee join replacement [50], an ^18^F-NaF tracer was used to set a baseline to evaluate implant stability based on the osteoblast and osteoclast activity and related presence of bone minerals. 

However, even if the radiotracer does not interact with the implant, the gamma photons that are the basis of the detection are influenced by the presence of foreign matter, resulting in additional absorption, attenuation, deflection and scatter. To illustrate this, Table 3 lists the linear attenuation values for biodegradable implant materials at the energy level of emitted gamma photons. Because general image reconstruction algorithms are designed to work on the basis of natural body tissues, the implants cause the appearance of image artefacts, mostly due to the use of CT attenuation maps [51,52]. To deal with this, similar to CT and MRI protocols, implants are recommended to be oriented along the *x*-axis of the scan and to have a uniform shape without sharp angles. 

### 2.3. Ultrasound Imaging

As one of the most safe, comfortable and simple imaging modalities, ultrasound is widely used to study structures and physical processes happening under the cover of the soft tissues. Ultrasound imaging uses the principle of sound waves echoing from the borders of mechanically different tissues [53], due to the variation of acoustic impedance. At the same time, the higher the sound frequency, the higher is the resolution but lower the penetration depth. Increased energy increases the imaging depth, however that can lead to side effects such as heating, acoustic cavitation and acoustic streaming [54]. The heating is one of the primal concerns associated with implant imaging, as the healing process can be by thermal damage [55]. 

Acoustic impedance is defined as material density (Table 1) and sound velocity in that material. The larger the difference between two tissues, the higher is the number of sound waves that are reflected, and the clearer is the image border. Considering that a significant number of orthopaedic implants is inside the bone, there are at least two acoustic impedance borders in between—from muscle to bone and from bone to implant. As a result, only a very limited amount of echo signal from implant within the bone can reach the receiver. Taking into account the attenuation, scatter, reflection and all the noise, ultrasound is of limited use for imaging such implants. Furthermore, it is important that both density and sound velocity are heavily dependent on the structure and composition of the materials, which can significantly differ even in similar alloys, composites and polymers. 

Although US is limited to soft tissues and the topography of hard surfaces [56], it still is a suitable tool for non-invasive and low-discomfort monitoring of the conditions outside the bone inner volume. That allows to evaluate the external aspect of bone and external implants such as fixation plates, monitor the wound healing and the inflammation process at the implantation site and the healing of bone fractures [57]. For biodegradable implants, US is suitable to observe the changes in surface structures caused by implant degradation and bone growth. It is also used to monitor soft tissue and bone healing and to evaluate the state of the inflammation at the wound site based on the measurements of accumulated liquid and oedema. 

### 2.4. Photoacoustic Imaging

Photoacoustic imaging (PAI) relies on the optical absorbance qualities of the tissues and included optical contrast agents down to molecular level. The target chromophores absorb the specific wavelength laser pulses, and the optical energy is converted into detectable sound pressure waves. These chromophores can be endogenous (free and bound water, oxyhaemoglobin, deoxyhaemoglobin, melanin, lipids) and exogenous (mostly small molecule dyes—indocyanine green, Methylene Blue Dye, nanoparticles, designed reporter gene agents etc.) [58].

**Figure 7:**
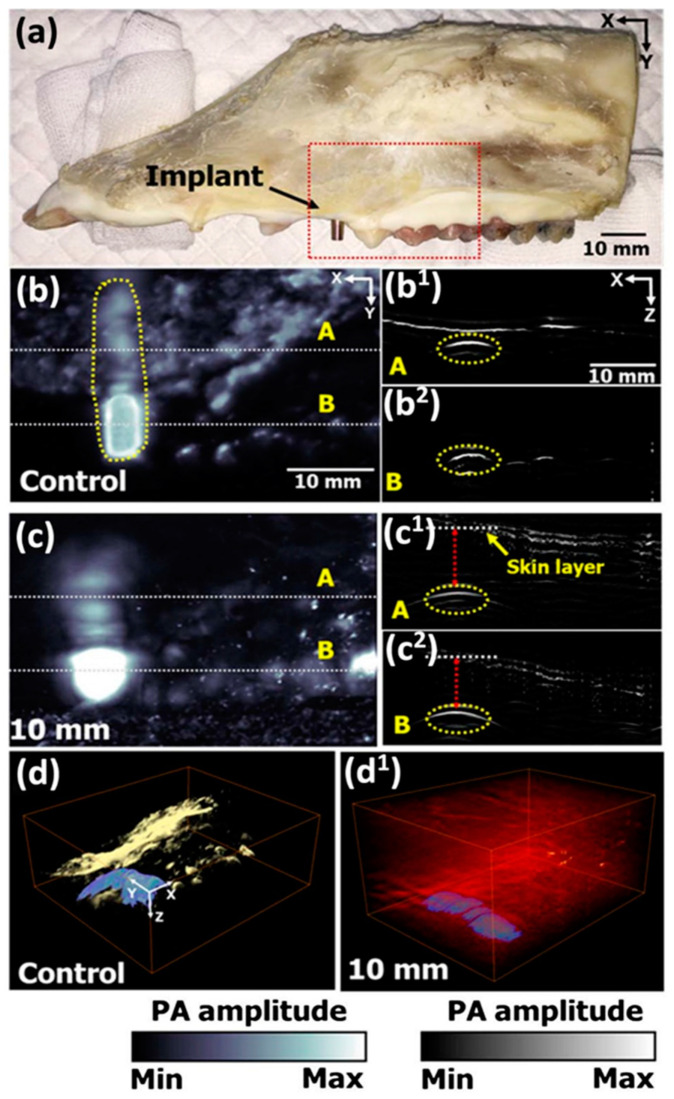
Ex vivo PA image of porcine jawbone with titanium implant abutment (EZ Post, MIEP3525HT, Megagen, Korea) and a fixture (MIIF3008C, Megagen, Korea). (**a**) Jawbone specimen. (**b**) PA image at 1064 nm excitation. (**c**) PA MAP images from (**b**) under 10 mm of chicken tissue. (**d**) 3D render of bare bone specimen (**d**) and under 10 mm chicken tissue (**d^1^**). (**b^1^**,**b^2^**) and (**c^1^**,**c^2^**) are cross-sectional PA images of the dashed line areas in corresponding (**b**,**c**) images.Reprinted with permission form Lee et al. (2017) [59] Copyright The Optical Society of America, 2017.

The use of PAI for implant monitoring has been previously explored in studies such as by Lee et al. [59] (Figure 7), who achieved reasonable ability to distinguish titanium implant covered by bone or meat, at depths relevant for dentistry applications. However, the depth penetration limit and noticeable optical attenuation makes it increasingly difficult to image the targets that are located deeper than 10–20 mm, limiting the clinical application of PAI to targets near the surface [60]. At the same time, PAI resolution is dependent on the depth based on “factor of 200 rule of thumb”, with resolution being 1/200th of the depth [61,62]. For preclinical applications, where small animal studies are prevalent, PAI is less limited by depth, and can be used to gather molecular data from the whole target area [63]. 

Photoacoustic imaging also has potential to be used with biodegradable implants. The possibility to observe in vivo the molecular activity during implant degradation is one of the desired tools in the relevant research. Notably, photoacoustic measurements of oxyhaemoglobin and deoxyhaemoglobin levels reflect the state of blood supply and angiogenesis in the wound area. 

### 2.5. Magnetic Resonance Imaging

MRI is one of the most advanced, non-invasive and low-discomfort imaging modalities, which is only limited by high costs, personnel qualification level and incompatibility with ferromagnetic materials. MRI is based on nuclear magnetic polarization created through static magnetic field (*B*_0_), which is disturbed with a pulse of radiofrequency (RF) field at Larmor frequency (f_o_), which in turn is calculated based on the strength of magnetic field and gyromagnetic ratio (γ) of the targeted nucleus or particle (with formula being f_o_ = γ × *B*_0_) [53]. The resulting disturbance in equilibrium is measured as alternating voltage in surrounding detection coils. The speed at which the disturbed magnetic polarization goes through the process of normalization can be used to differentiate the condition of the tissues and reconstruct this as a visual image.

MRI is best at imaging soft tissues and liquids, because of the high content of H^+^ protons [64]. This makes it suitable for imaging inflammation through gadolinium contrast [65] or by detecting liquid accumulations such as oedema and synovitis. Similarly, MRI is actively used to study vascularization through blood flow quantification analysis using perfusion MRI techniques such as arterial spin labelling (ASL), dynamic susceptibility contrast (DSC), dynamic contrast enhanced (DCE), and intravoxel incoherent monition (IVIM) tools [66]. 

Due to the operating principle of MRI, hard objects without free H^+^ protons, like bones and implants, are not optimal imaging targets. However, with bone still being living tissue, it remains possible to image osteolysis at the damage sites [67], giving a clear view of the borders. It is also possible to detect non-ferromagnetic implants such as poly(lactic-co-glycolic acid) (PLGA), tricalcium phosphate (TCP) and polylactic acid (PLA) based on their negative contrast [68,69] and that should carry over to other biodegradable ceramic and polymer implants. Some materials such as glass and plastics are known to be diamagnetic—magnetized in the direction opposite to the magnetic field. However, there is no data available about diamagnetic biodegradable implant materials such as bioglass and polymers [70]. While magnesium and zinc are also non-ferromagnetic, their difference in magnetic susceptibility from surrounding tissues still produces a low level of imaging artefacts and geometric distortions [38,71]. Iron, as a strongly magnetic material, makes implants unsuitable for MRI imaging.

## 3. Discussion and Conclusions

Due to the ever-expanding list of imaging targets and the constant refinement of imaging technologies, it is becoming increasingly complex to choose a single appropriate modality. Depending on variables such as implant material, surrounding tissue and placement depth, the same target may require different imaging techniques or a combination of them. Thanks to the advances in composite material development, it is possible to produce implants that have drastically different properties. 

On one hand, it improves the biocompatibility of composites, on the other hand, these composites are often patented. This results in competing manufacturers producing their own alternatives and patenting these in turn. Hence, there are many new materials, often with severely lacking information about their composition and even some basic physical properties. This further complicates imaging studies, since without knowing these properties, it is more difficult and time-consuming to design and perform the studies. For the same reasons, the information about some modalities may be underrepresented compared to others. For example, because X-ray and CT have been established as the gold standard in multiple areas of material and biomedical studies, it was possible to provide material attenuation data relevant for the modality. At the same time, information about magnetic susceptibility, sound velocity and absorption spectrum (for MRI, US and PAI) for biodegradable materials was insufficient for an adequate compilation. It is necessary for implant material studies to put more focus on reporting these characteristics.

To improve the quality of the data obtained using imaging, one of the approaches is to design implants that consider the specifics of the imaging modality. With the developing technologies of custom production such as 3D printing, this is a possible future for preclinical studies that are aiming to understand the details of the body and implant interactions. As an example, it is possible to include chromophores to the bulk material, producing an implant whose degradation kinetics can be followed with PAI at the molecular level. However, such an approach is unsuitable for clinical use and for studying the already existing materials. This is where the multimodal imaging can be of advantage. By achieving an optimal combination of imaging techniques that help to cancel out or mitigate the individual shortcomings, the diagnosis, evaluation and treatment can be done with the highest accuracy and reliability.

When making use of multimodal imaging, the imaging techniques need to be chosen based on how suitable they are for the intended combination of the target(s) and the aim(s). As an example, angiogenesis is a common and reliable imaging biomarker for evaluating tissue healing, because new blood vessels are required to support the growth and functioning of the new tissues. It can be imaged using all imaging modalities mentioned in this paper (CT, PET, US, PAI and MRI). By way of example, using PET, the angiogenetic process (i.e. new blood vessel formation associated to vascular endothelium proliferation) can be studied non-invasively by assessing the regulation of the integrin expression (among the integrin superfamily the α_V_β_3_ isoform is the most used). The α_V_β_3_ expression is quantified using a tracer such as ^68^Ga-NODAGA-RGD [72,73] and is one of the few ways to produce images of in vivo metabolism. The drawback of using PET is limited resolution (1–2 mm), which, while rarely crucial in clinical studies, can be a limiting factor for small animal studies due to the scaling. However, PET is commonly combined with CT and also with MRI, which have well established angiography techniques, allowing us to image vasculature in high detail. At the same time, the different modalities allow us to better visualise the borders between the implant and the tissues, which is also usable for artefact correction. Alternatively, because PET has necessary intervals between imaging, using different tracers to image the same target is problematic. In such cases, PAI can be utilised to image α_V_β_3_ expression, and PET to follow bone mineralization with the help of ^18^F-NaF. 

Table 4 combines the previously mentioned imaging modalities and their suitable imaging targets that have been reported in the literature and can be applied for implant studies.

While histology, as the traditional gold standard for evaluating biological changes, is capable of providing the most detailed analysis and validation of the target conditions, it has severe shortcomings due to its invasive and destructive procedure which is limited to a singular time point [66]. The changes in tissue remodelling are interlinked with the structural and chemical changes of the implants, producing an increasingly complex web of multi-level interactions from molecular to functional levels that keep changing over time. As the tissue repair and remodelling progress, the priority properties of implants change accordingly—from the importance of biocompatibility at the initial stage, to tissue integration, bulk degradation rate and mechanical stability at the later stages. With the necessary requirement to acquire longitudinal data in vivo while maintaining the necessary level of detail, the modern approach is to integrate multiple imaging techniques and use a wide range of available target probes (tracers, contract agents, chromophores etc.) and specialized settings (such as T1/T2 imaging in MRI or Doppler in US).

As mentioned before, different imaging modalities show different variable properties of the imaging target—CT differentiates the attenuation coefficient of the matter, which is linked to density values, PET targets specific metabolic activities, US is for separating tissues based on their difference in acoustic impedance, USPA makes use of optical absorbance differences of molecules and MRI makes use of the magnetic properties of the H^+^ protons and how quickly the disturbed magnetic polarization of nuclei returns to normal (therefore, image differences can be related to water content and how it is bound in tissues). The process of imaging is directly followed by image processing and analysis, the procedures that translate the visual information into quantitative and qualitative parameters. The use of mathematical comparison and evaluation through statistical methods confirms the validity of the results and allows the translation of the data between the similar cases. In implant and tissue imaging, the texture analysis is essential, and can be interpreted as classifying different images or image regions into distinct groups [87]. Most image processing software has segmentation toolkits, facilitating the manual separation of image regions, and diagnostic procedures in, for example, radiology have stably adapted the use of computer-aided diagnosis (CAD) [88]. For tissue study, it is important to distinguish the individual tissues, organs and regions of interest (ROI), such as the wound site or pathology. Observation of biodegradable implants requires an accurate localization of the implant and its borders, especially because of the constant changes over time. There lies the challenge of unclear border areas and artefacts, where individual judgement of the operator doing the analysis can affect the resulting data [89]. The use of machine learning, such as deep neural networks [90,91], has a potential to improve the speed and accuracy of image analysis, reducing the effect of human error and differences in individual judgements. The automatic learning of useful representations and features makes it efficient and flexible, increasing the range of uses in the biomedical field to also include image acquisition and reconstruction processes [91]. 

It can be concluded that the multimodal and multi-parametric imaging can provide all the complimentary information and longitudinal views that are necessary in modern biomaterial research. To allow unhindered progress of research and further development, it is necessary to have a flow of information and continuous cooperation between the fields of imaging, engineering and biomedicine. Considering the wide range of involved specializations, this information needs to be easy to access and understand. Be it for translating the results “from bench to bedside” or to ensure high selectivity and sensitivity of the target data, having clear guidelines is helpful for all involved parties. This work provided an overview of the techniques that can be considered to be most suitable for imaging the processes that involve biodegradable implants. By further developing the optimal approaches for different implants and imaging targets, the resulting standardization and accumulated knowledge will promote the scientific and technological development in the field of biomaterial research.

## Figures and Tables

**Table 1:** Biodegradable implant materials reported in the literature, the main constituent atoms and average density [18,19,20,21,22,23,24,25,26,27,28,29,30,31,32].

		Main Atoms	Density
**Ceramics**			
Calcium phosphates			
Hydroxyapatite		Ca, P	3.1–3.2
Dicalcium phosphate dihydrate	DCPD	Ca, P	2.3
	Brushite	Ca, P	2.3–2.33
Tricalcium phosphate	TCP	Ca, P	3
Tetracalcium phosphate	TeCP	Ca, P	3.06
Bioglass		Si, Ca, Na, O	2.6–2.7
**Polymers**			
Poly(amino acids)	e.g., poly(γ-benzyl-L-glutamate)	C, H	1.2
Poly(ortho esters)		C, O, H	0.39–0.46
Polyphosphazenes		P, N	
Poly(propylene fumarate)		C, O	0.998
Polyesters			
Aliphatic	Polylactide	C, H, O	1.25–1.27
	Polyglycolide	C, H, O	1.5–1.6
	Copolymers		
Polyhydroxy- alkanoates	Polyhydroxy- butyrate	C, H, O	1.18–1.26
	Polyhydroxy- valerate	C, H, O	
	Polyhydroxy- hexanoate	C, H, O	
	Polyhydroxy- octanoate	C, H, O	
	Copolymers		
Polycaprolactone		C, H, O	1.10–1.15
Polydioxanone		C, O	1.318
**Metals**			
Magnesium	Alloyed with Ca, Zn, Al, rare metals	Mg	1.738
Iron		Fe	7.874
Zinc		Zn	7.140
**Body tissues**			
Bone (cortical)			1.9
Muscle (skeletal)			1.06

**Table 2:** Calculated linear attenuation coefficients (cm^−1^) for biodegradable implant materials (magnesium and hydroxyapatite), titanium, cortical bone and skeletal muscle from to 5 to 150 keV. Values can vary depending on structure and density.

Photon Energy (keV)	Mg	Ti	Hydroxyapatite	Cortical Bone	Skeletal Muscle
5	273.21	3073.09	967.20	350.59	44.08
6	161.41	1941.18	593.03	213.70	25.47
7.112					15.23
7.112					15.25
8	69.36	906.61	270.07	96.65	10.67
9.659				56.85	6.03
9.659				56.89	6.04
10	35.70	494.76	144.49	51.53	5.44
15	10.55	159.02	45.20	16.10	1.65
20	4.48	69.62	19.59	7.03	0.78
30	1.44	21.42	6.11	2.29	0.35
40	0.74	9.35	2.81	1.13	0.25
50	0.50	5.04	1.65	0.72	0.22
60	0.39	3.14	1.13	0.54	0.20
80	0.31	1.63	0.73	0.39	0.18
100	0.27	1.10	0.58	0.34	0.17
120	0.25	0.86	0.50	0.31	0.16
140	0.24	0.73	0.46	0.28	0.16
150	0.23	0.68	0.44	0.28	0.15

**Table 3:** Biodegradable implant materials and their linear attenuation coefficients (cm^−1^) of 511 keV photons.

Type	Material	Attenuation Coefficient at 511 keV
Metals	Mg	0.15
	Fe	0.64
	Zn	0.58
	Ti	0.36
Ceramics	Hydroxy- apatite	0.27
	DCPD	0.20
	Brushite	0.21
	TCP	0.27
	TeCP	0.27
	Bioglass	0.23
Polymers	poly(γ-benzyl-L-glutamate)	0.11
	Polypropylene fumarate	0.11
	Polylactide	0.11
	Poly- glycolide	0.14
	Polyhydroxy-butyrate	0.11
	Polyhydroxy-valerate	0.11
	Polycapro- lactone	0.11
	Poly- dioxanone	0.12
Tissues	Cortical bone	0.17
	Skeletal muscle	0.10

**Table 4:** Preclinical imaging modalities, their properties and application in relation to biodegradable implants.

	CT	PET	US	PAI	MRI
**Depth**	- [72]	- [72]	>50 mm [74]	>50 mm [60]	-
**Spatial resolution**	0.05 mm [75]	1–2 mm [72]	Up to 0.15 mm at 25 MHz [76]	1/200th of depth [60,61]	>0.1 mm [77]
**Target**					
**Bone tissue**	Implants and tissues of different attenuation	Molecular activity with target tracers	Surface topography	Surface topography	Unsuitable
**Soft tissues**		Molecular activity with target tracers	Structures and borders	Structures and molecular chromophores	Highly efficient
**Implant**			Surface topography	Mostly surface topography	Unsuitable
**Biomarkers**					
**Inflammation**	Visual oedema and liquid accumulation, liquid iodine contrast [41]	e.g., ^18^F-FDG [78]	Visual oedema and liquid accumulation	Oxygen level dynamics [79]	Visual oedema and liquid accumulation
**Bone healing**	Visual changes	e.g., ^18^F-NaF [44, 72.73]	Exceptional for early ossification [57]	Chromophores; tissue morphology changes during remodelling	Contrast of low signal from bone with high signal from soft tissues [80]
**Angiogenesis**	blood vessels with iodine contrast [81]	e.g., ^68^Ga-NODAGA-RGD [47]; ^68^Ga-NOTA-RGD [47,82]; ^68^Ga-DOTA-E[c(RGDfK)]_2_ [83]	Vascular imaging using microbubbles and binding agents [84,85]	Integrin-binding chromophores; blood oxygenation	e.g., Gadolinium contrast, perfusion capacity [86]
**Advantages**	Simple, fast	Large choice of radiotracers for different targets, possible to design tracers for specific targets; metabolic data	Simple; non-invasive; no contraindications	Can image molecular activity; differentiate targets based on absorption spectra	High resolution and depth; very good soft tissue differentiation; possible to use contrast agents
**Disadvantages**	Radiation; requires high attenuation between target and surroundings; contrast agents require injections; implant artefacts [35,36,37,40,78]	Radiation; high price; complexity; injections; long duration; requires either in-house isotope production and tracer laboratory or be within transportation range	Limited depth; can’t image inside hard objects	Very limited depth; light absorption; complexity; exogenous contrast agents require injections	High price; complexity; unsuitable for ferromagnetic implants [35]; poor imaging of targets lacking H^+^ protons; better resolution requires longer imaging time; contrast agents are injected

## Data Availability

Not applicable.

## Appendix A

**Table A1:** Calculated total attenuation coefficients without coherent scattering for biodegradable implant materials, titanium, cortical bone and skeletal muscle from to 5 to 150 keV.

Total Attenuation without Coherent Scattering cm^2^/g																				
	Metals				Ceramics						Polymers								Tissues	
keV	Mg	Fe	Zn	Ti	Hydroxy-Apatite	DCD	Brushite	TCP	TeCP	Bioglass	Poly (γ-Benzyl-l-Glutamate)	Poly-Propylene Fumarate	Poly- Lactide	Poly-Glycolide	Poly- Hydroxy-Butyrate	Poly- Hydroxy-Valerate	Poly-Capro-Lactone	Poly-Dioxanone	Cortical Bone	Skeletal Muscle
5	157.20	137.40	208.70	682.00	312.00	217.60	217.60	309.30	329.80	207.80	24.68	30.31	30.39	33.85	28.07	26.39	25.13	31.08	182.60	41.98
6	92.87	82.78	126.40	430.80	191.30	132.60	132.60	189.60	202.40	125.90	14.11	17.37	17.42	19.42	16.08	15.11	14.38	17.82	111.30	24.26
7.112		51.45																		14.50
7.112		406.00																		14.52
8	39.91	304.10	56.75	201.20	87.12	59.89	59.89	86.28	92.34	56.34	5.83	7.18	7.21	8.04	6.66	6.26	5.96	7.38	50.34	10.16
9.659			33.45																29.61	5.74
9.659			252.00																29.63	5.75
10	20.54	169.50	231.50	109.80	46.61	31.88	31.88	46.14	49.45	29.80	2.96	3.64	3.66	4.07	3.37	3.18	3.02	3.74	26.84	5.18
15	6.07	56.34	80.24	35.29	14.58	9.92	9.92	14.42	15.49	9.19	0.93	1.12	1.13	1.24	1.05	0.99	0.95	1.15	8.39	1.57
20	2.58	25.17	36.55	15.45	6.32	4.31	4.31	6.25	6.71	3.97	0.48	0.54	0.55	0.59	0.52	0.50	0.49	0.56	3.66	0.74
30	0.83	7.89	11.71	4.75	1.97	1.38	1.37	1.95	2.09	1.26	0.26	0.27	0.28	0.28	0.27	0.27	0.26	0.28	1.19	0.33
40	0.43	3.45	5.16	2.08	0.91	0.66	0.66	0.90	0.95	0.61	0.21	0.21	0.21	0.22	0.21	0.21	0.21	0.21	0.59	0.24
50	0.29	1.83	2.74	1.12	0.53	0.41	0.41	0.53	0.55	0.38	0.18	0.18	0.19	0.19	0.20	0.19	0.19	0.19	0.38	0.21
60	0.22	1.11	1.65	0.70	0.36	0.30	0.30	0.36	0.38	0.28	0.18	0.18	0.18	0.18	0.18	0.18	0.18	0.18	0.28	0.19
80	0.18	0.54	0.77	0.36	0.24	0.21	0.21	0.23	0.24	0.20	0.17	0.16	0.17	0.16	0.17	0.17	0.17	0.17	0.20	0.17
100	0.16	0.33	0.45	0.24	0.19	0.18	0.18	0.18	0.19	0.17	0.16	0.15	0.16	0.15	0.15	0.16	0.16	0.16	0.18	0.16
120	0.14	0.24	0.31	0.19	0.16	0.16	0.16	0.16	0.16	0.15	0.15	0.14	0.15	0.14	0.15	0.15	0.16	0.15	0.16	0.15
140	0.14	0.19	0.24	0.16	0.15	0.15	0.15	0.15	0.15	0.14	0.14	0.14	0.14	0.14	0.15	0.15	0.15	0.15	0.15	0.15
150	0.13	0.18	0.21	0.15	0.14	0.14	0.14	0.14	0.14	0.14	0.14	0.14	0.14	0.14	0.15	0.14	0.15	0.14	0.15	0.14
